# High virulence does not necessarily impede viral adaptation to a new host: a case study using a plant RNA virus

**DOI:** 10.1186/s12862-017-0881-7

**Published:** 2017-01-19

**Authors:** Anouk Willemsen, Mark P. Zwart, Santiago F. Elena

**Affiliations:** 1 Instituto de Biología Molecular y Celular de Plantas (IBMCP), Consejo Superior de Investigaciones Científicas-Universidad Politécnica de Valencia, Campus UPV CPI 8E, Ingeniero Fausto Elio s/n, 46022 València, Spain; 2 Present address: MIVEGEC (UMR CNRS 5290, IRD 224, UM), National Center for Scientific Research (CNRS), 911 Avenue Agropolis, BP 64501, 34394 Cedex 5 Montpellier, France; 3 Present address: Institute of Theoretical Physics, University of Cologne, Zülpicher Straße 77, 50937 Cologne, Germany; 4 The Santa Fe Institute, 1399 Hyde Park Road, Santa Fe, NM 87501 USA

**Keywords:** Adaptation, Experimental evolution, Genome architecture evolution, Host-pathogen interactions, Virulence, Virus evolution

## Abstract

**Background:**

Theory suggests that high virulence could hinder between-host transmission of microparasites, and that virulence therefore will evolve to lower levels. Alternatively, highly virulent microparasites could also curtail host development, thereby limiting both the host resources available to them and their own within-host effective population size. In this case, high virulence might restrain the mutation supply rate and increase the strength with which genetic drift acts on microparasite populations. Thereby, this alternative explanation limits the microparasites’ potential to adapt to the host and ultimately the ability to evolve lower virulence. As a first exploration of this hypothesis, we evolved *Tobacco etch virus* carrying an eGFP fluorescent marker in two semi-permissive host species, *Nicotiana benthamiana* and *Datura stramonium*, for which it has a large difference in virulence. We compared the results to those previously obtained in the natural host, *Nicotiana tabacum*, where we have shown that carriage of *eGFP* has a high fitness cost and its loss serves as a real-time indicator of adaptation.

**Results:**

After over half a year of evolution, we sequenced the genomes of the evolved lineages and measured their fitness. During the evolution experiment, marker loss leading to viable virus variants was only observed in one lineage of the host for which the virus has low virulence, *D. stramonium*. This result was consistent with the observation that there was a fitness cost of *eGFP* in this host, while surprisingly no fitness cost was observed in the host for which the virus has high virulence, *N. benthamiana*. Furthermore, in both hosts we observed increases in viral fitness in few lineages, and host-specific convergent evolution at the genomic level was only found in *N. benthamiana*.

**Conclusions:**

The results of this study do not lend support to the hypothesis that high virulence impedes microparasites’ evolution. Rather, they exemplify that jumps between host species can be game changers for evolutionary dynamics. When considering the evolution of genome architecture, host species jumps might play a very important role, by allowing evolutionary intermediates to be competitive.

**Electronic supplementary material:**

The online version of this article (doi:10.1186/s12862-017-0881-7) contains supplementary material, which is available to authorized users.

## Background

From both applied and fundamental perspectives, virulence is a key phenotypic trait of microparasites. In medicine and agriculture, it is crucial to understand mechanistically how microparasites harm the host, in order to devise effective interventions. From a more fundamental perspective, evolutionary biologists have long been interested in understanding why many microparasites are highly virulent. It has been suggested that virulence reduces between-host transmission, and that selection would therefore act to maximize between-host transmission by reducing virulence [1, 2]. High virulence would signal maladaptation, for example following a host-species jump, and eventually be selected against. The ubiquity of microparasitic virulence and the fact that many apparently well-adapted microparasites have high virulence led to a more sophisticated framework: the hypothesis that there are tradeoffs between virulence and transmission [2–5]. This framework posits that high levels of replication could increase the probability of a microparasite being transferred to a new host, whilst also increasing the probability that the host would die quickly and the temporal window for transmission would be very brief. Under this more plausible framework, virulence evolves to the level that optimizes between-host transmission [4, 6, 7].

The tradeoff hypothesis forms the cornerstone for theoretical frameworks considering the evolution of virulence in many different pathosystems. Many important additions to the framework have been made, for example recognizing that within-host competition and opportunism can lead to increases in virulence [8–10]. Moreover, the importance of other factors at the between-host level have been given consideration, such as self-shading [11]. The effects of evolution on microparasitic virulence have therefore been given considerable attention, although the number of experimental studies that address this issue is still rather limited, especially for viruses [12].

Conversely, virulence itself could also have profound effects on evolution, including its own evolutionary dynamics [13]. This reversed causality is already apparent from the tradeoff model, under which microparasites with suboptimal virulence will undergo reduced between-hosts transmission. All else being equal, if a smaller number of hosts are infected, effective population size will be decreased, increasing the strength of genetic drift and decreasing the mutation supply rate. In addition, the evolution to optimum virulence may be slow as this optimum is not static and can shift towards lower virulence as the density of susceptible hosts decreases [14]. Moreover, a wide range of virulence can be associated with each step of evolution towards the optimum, where selection favors genotypes with higher fitness that may improve transmission but not necessarily improve virulence [13]. Besides these effects of virulence on evolution, it is conceivable that a similar within-host effect could also occur, when virulence curtails host development (i.e., host growth or evolution) and thereby limits the host resources available to the microparasite. Virulence would then limit the microparasite effective population size within hosts, again reducing the mutation supply and thereby slowing the rate of adaptation. Interestingly, (*i*) a low effective population size, (*ii*) a non-static optimum virulence, (*iii*) large variations in virulence on the fitness landscape, and (*iv*) reduced host-development, could limit the rate at which lower virulence evolves, meaning that high virulence might persist longer than suggested by the simple tradeoff model [13].

There are many reasons why high virulence in host-pathogen interactions could emerge, but the most likely avenue is probably a change of host species. For example, infection of Ebola virus in bats is asymptomatic, while in humans and other primates the death rate is high [15]. Changes in virulence have been explained by the host phylogeny, where similar levels of virulence are displayed by closely related hosts and host jumps across large genetic distances may result in high virulence [16]. However, if a microparasite is confronted with a new host environment in which its level of virulence is altered, how does virulence affect its ability to adapt to the new host?

Here we address this question using *Tobacco etch virus* (TEV; genus *Potyvirus*, family *Potyviridae*), a (+)ssRNA virus with a 9.5 kb genome that infects a wide-range of host plants, and an experimental evolution approach. To consider the effect of virulence (e.g., virus-induced host mortality or reduction in biomass) on virus adaptation, we looked for two natural host species in which (*i*) there was some evidence that TEV potential for adaptation would be roughly similar, and (*ii*) there was a large difference in virulence. The distribution of mutational fitness effects (DMFE) of TEV has been compared in eight host species, and this study concluded that there were strong virus genotype-by-host species interactions [17]. For many host species distantly related to the natural host of TEV, *Nicotiana tabacum*, the DMFE changed drastically; many mutations that were neutral or deleterious in *N. tabacum*, became beneficial. However, for two related host species, *Nicotiana benthamiana* and *Datura stramonium*, most mutations tested remained neutral or deleterious [17], implying that the fraction of beneficial mutations in both hosts is small. Moreover, virus accumulation after one week of infection is also similar for both hosts [18]. On the other hand, TEV infection of *N. benthamiana* will typically result in heavy stunting and the death of the plant within a matter of weeks, whereas TEV infection of *D. stramonium* is virtually asymptomatic. These symptoms are very different from symptoms observed for TEV in its natural host *N. tabacum*, which does not lead to plant death but consist of vein clearing, mosaic mottling, chlorosis, and stunting [19, 20]. Whilst there are many similarities between TEV infections in these two hosts, one key difference is host-pathogen interactions and therewith levels of viral virulence brought about.

**Fig. 1:**
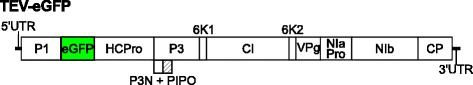
Schematic representation of TEV-eGFP. The *eGFP* gene is located between *P1* and *HC-Pro* genes. Proteolytic cleavage sites were provided at both ends of eGFP

As a first exploration of the effects of virulence on microparasite evolution, we therefore decided to evolve TEV in *N. benthamiana* and *D. stramonium*. By serially passaging each independent lineage in a single plant every round, our study maximizes within-host selection. This setup allows us to exclusively focus on the effects of within-host selection, although for our model system we expect to see large differences in the resulting population size and the extent of virus movement within the host. Moreover, to immediately gauge whether adaptive evolution might be occurring, we passaged a TEV variant expressing a marker protein (Fig. 1), the enhanced GFP (eGFP). This exogenous sequence of 762 nts increases TEV genome to a size just over 10 kb. Upon long-duration passages in *N. tabacum*, the *eGFP* gene is quickly lost due to its strong fitness cost, and its loss is reliably indicated by a loss of eGFP fluorescence [21]. Therefore, we can use the time to *eGFP* loss in the virus population as a real-time indicator of adaptation. To determine the frequency of *eGFP* loss and host-specific mutations in each viral population (i.e., lineage), the ancestral virus and evolved lineages were sequenced by Illumina technology. According to the above hypothesis that high virulence may impair the rate of microparasite evolution, we expect that adaptive evolution would occur more quickly in the host species for which TEV has lower virulence, *D. stramonium*, than in the host species for which it has high virulence, *N. benthamiana*. Hence, we expected that in *D. stramonium* (*i*) the eGFP marker would be lost more rapidly, (*ii*) there would be more sequence-level convergent evolution, and (*iii*) there would be larger increases in within-host competitive fitness. However, our experiments did not confirm these simple hypotheses. First of all, the loss of *eGFP* could not be used as an indicator of adaptation in neither *D. stramonium* nor *N. benthamiana*. Second, host-specific convergent evolution at the genomic level was only found in *N. benthamiana*. Third, in both hosts we observe only one evolved lineage with increases in within-host competitive fitness, and surprisingly the *eGFP* marker does not seem to have a fitness cost in *N. benthamiana*. These results were unexpected based on our previous work in *N. tabacum* [21], but they do exemplify the extent to which a host species jump can be a game changer for RNA virus evolutionary dynamics.

## Methods

### Virus stocks, plants and serial passages

The TEV genome used to generate TEV-eGFP virus was originally isolated from *N. tabacum* plants [22]. To generate a virus stock of the ancestral TEV-eGFP, the pMTEV-eGFP plasmid [23] was linearized by digestion with *Bgl*II prior to in vitro RNA synthesis using the mMESSAGE mMACHINE® SP6 Transciption Kit (Ambion), as described in [24]. The third true leaf of 4-week-old *N. tabacum* L var Xanthi *NN* plants was mechanically inoculated with 5 μg of transcribed RNA. All symptomatic tissue was collected 7 days post-inoculation (dpi).

For the serial passage experiments, 500 mg homogenized stock tissue was ground into fine powder and diluted in 500 μl phosphate buffer (50 mM KH_2_PO_4_, pH 7.0, 3% polyethylene glycol 6000). From this mixture, 20 μl were then mechanically inoculated on the sixth true leaf of 4-week old *N. benthamiana* Domin plants and on the third true leaf of 4-week old *D. stramonium* L plants. Ten independent replicates were used for each host plant. Based on a previous study done in *N. tabacum* [21], passages of TEV-eGFP in *D. stramonium* were done every 9 weeks. In *N. benthamiana* the virus induces host mortality, and therefore the passages had to be restricted to 6 weeks for this host. At the end of the designated passage duration all leaves above the inoculated one were collected and stored at −80 °C. For subsequent passages the frozen tissue was homogenized and a sample was ground and resuspended with an equal amount of phosphate buffer [21]. Then, new plants were mechanically inoculated as described above. Three 9-week passages were performed for lineages evolved in *D. stramonium* (27 weeks of evolution) and five 6-week passages for lineages evolving in *N. benthamiana* (30 weeks of evolution), making the total time of evolution similar in both hosts. For both *D. stramonium* and *N. benthamiana*, all individual plants used were grown from large stocks of seeds collected from self-pollinated individual plants, therefore all plants for each host were of identical genotype. All plants were kept in a biosafety level 2 greenhouse at 24 ° C with 16 h light:8 h dark photoperiod.

### Reverse transcription polymerase chain reaction (RT-PCR)

To determine whether deletions occurred at the *eGFP* locus, RNA was extracted from 100 mg homogenized infected tissue using the InviTrap Spin Plant RNA Mini Kit (Stratec Molecular). Reverse transcription (RT) was performed using MMuLV reverse transcriptase (Thermo Scientific) and reverse primer 5’-CGCACTACATAGGAGAATTAG-3’ located in the 3’UTR of the TEV-eGFP genome (GenBank accession; KC918545, positions 10235–10255). PCR was then performed with Taq DNA polymerase (Roche) and primers flanking the *eGFP* gene: forward 5’-GCAATCAAGCATTCTACTTC-3’ (positions 48–67), and reverse 5’-CCTGATATGTTTCCTGATAAC-3’ (positions 2530–2550). PCR products were resolved by electrophoresis on 1% agarose gels.

### Illumina sequencing, variants, and SNP calling

For Illumina next-generation sequencing (NGS) of the evolved and ancestral lineages, the viral genomes were amplified by RT-PCR using AccuScript Hi-Fi (Agilent Technologies) reverse transcriptase and Phusion DNA polymerase (Thermo Scientific), with six independent replicates that were pooled. Each virus was amplified using three primer sets, generating three amplicons of similar size (set 1: 5’-GCAATCAAGCATTCTACTTCTATTGCAGC-3’ and 5’-CCTGATATGTTTCCTGATAAC-3’ (positions 48–76 and 2530–2550); set 2: 5’-ACACGTACTGGCTGTCAGCG-3’ and 5’-CATCAATGTCAATGGTTACAC-3’ (positions 2217–2236 and 5324–5344); set 3: 5’-CCCGTGAAACTCAAGATAG-3’ and 5’-CGCACTACATAGGAGAATTAG-3’ (positions 5044–5062 and 10235–10255). Equimolar mixtures of the three PCR products were made. Sequencing was performed at GenoScreen (Lille, France: www.genoscreen.com). Illumina HiSeq2500 2 × 100 bp paired-end libraries with dual-index adaptors were prepared along with an internal PhiX control. Libraries were prepared using the Nextera XT DNA Library Preparation Kit (Illumina Inc.). Sequencing quality control was performed by GenoScreen, based on PhiX error rate and Q30 values.

Read artifact filtering and quality trimming (3’ minimum Q28 and minimum read length of 50 bp) was done using FASTX-Toolkit v.0.0.14 [25]. De-replication of the reads and 5’ quality trimming requiring a minimum of Q28 was done using PRINSEQ-lite v.0.20.4 [26]. Reads containing undefined nucleotides (N) were discarded. Initially, the ancestral TEV-eGFP sequence was mapped using Bowtie v.2.2.6 [27] against the reference TEV-eGFP sequence (GenBank accession: KC918545). Error correction was done using Polisher v2.0.8 (available for academic use from the Joint Genome Institute) and a consensus sequences was defined for the ancestral TEV-eGFP lineage. Subsequently, the cleaned reads of the evolved sequences were mapped using Bowtie v.2.2.6 against the new defined consensus sequence. Single nucleotide mutations for each viral lineage were identified using SAMtools’ mpileup [28] and VarScan v.2.3.9 [29], where the maximum coverage was set to 40000 and mutations with a frequency < 1% were discarded. Note that the single nucleotide mutations detected here can be fixed (frequency > 50%) in the evolved lineages, as the detection was done over the ancestral population. Hence, it allows us to compare the mutations that arose by evolving TEV-eGFP in the different hosts.

### Virus accumulation and within-host competitive fitness assays

Prior to performing assays, the genome equivalents per 100 mg of tissue of the ancestral virus stocks and all evolved lineages were determined for subsequent fitness assays. The InviTrap Spin Plant RNA Mini Kit (Stratec Molecular) was used to isolate total RNA of 100 mg homogenized infected tissue. Real-time quantitative RT-PCR (RT-qPCR) was performed using the One Step SYBR PrimeScript RT-PCR Kit II (Takara), in accordance with manufacturer instructions, in a StepOnePlus Real-Time PCR System (Applied Biosystems). Specific primers for the coat protein gene (*CP*) were used: forward 5’-TTGGTCTTGATGGCAACGTG-3’ (positions 9968–9987) and reverse 5’-TGTGCCGTTCAGTGTCTTCCT-3’ (positions 9998–10018). The StepOne Software v.2.2.2 (Applied Biosystems) was used to analyze the data. The concentration of genome equivalents per 100 mg of tissue was then normalized to that of the sample with the lowest concentration, using phosphate buffer.

For the accumulation assays, 4-week-old *N. benthamiana* and *D. stramonium* plants were mechanically inoculated with 50 μl of the normalized dilutions of ground tissue. Inoculation of each viral lineage was done on the same host plant on which it had been evolved, plus TEV and the ancestral TEV-eGFP virus on each of the hosts, using three independent plant replicates per lineage. Leaf tissue was harvested 10 dpi. Total RNA was extracted from 100 mg of homogenized tissue. Virus accumulation was then determined by means of RT-qPCR for the *CP* gene of the ancestral and the evolved lineages. For each of the harvested plants, at least three technical replicates were used for RT-qPCR.

To measure within-host competitive fitness, we used TEV carrying a red fluorescent protein: TEV-mCherry as a common competitor. This virus has a similar insert size and within-host fitness compared with TEV-eGFP [21]. All ancestral and evolved viral lineages were again normalized to the sample with the lowest concentration, and 1:1 mixtures of viral genome equivalents were made with TEV-mCherry [23]. The mixture was mechanically inoculated on the same host plant on which it had been evolved, plus TEV and the ancestral TEV-eGFP virus on each of the hosts, using three independent plant replicates per viral lineage. The plant leaves were collected at 10 dpi, and stored at −80 °C. Total RNA was extracted from 100 mg homogenized tissue. RT-qPCR for the *CP* gene was used to determine total viral accumulation, and independent RT-qPCR reactions were also performed for the mCherry sequence (GenBank accession: AY678264) using specific primers: forward 5’-CGGCGAGTTCATCTACAAGG-3’ (positions 360 to 379) and reverse 5’-TGGTCTTCTTCTGCATTACGG-3’ (positions 416–436). The ratio of the evolved and ancestral lineages to TEV-mCherry (*R*) is then *R*=(*n*_*CP*_-*n*_*mCherry*_)/(*n*_*mCherry*_), where *n*_*CP*_ and *n*_*mCherry*_ are the RT-qPCR measured copy numbers of *CP* and *mCherry*, respectively. Then we can estimate the within-host competitive fitness as $W=\sqrt[t]{R_t/{R}_o},$ where *R*_0_ is the ratio at the start of the experiment and *R*_*t*_ the ratio after *t* days of competition [24]. The statistical analyses comparing the fitness between lineages were performed using R v.3.2.2 [30] and IBM SPSS Statistics version 23.

## Results

### Experimental setup and fluorescent marker stability upon passaging of TEV-eGFP

TEV-eGFP was mechanically passaged in *N. benthamiana* and *D. stramonium*, as described in Zwart et al. 2014 [21]. In this previous study, we noted that 9-week long passages led to rapid deletion of *eGFP* as well as rapid convergent evolution in *N. tabacum* [21]. We therefore chose to use similar long passages to maximize the effects of selection in general, and in particular because we intended to use the loss of *eGFP* as a real time measure of adaptation. Although 9-week passages could be performed in *D. stramonium*, for *N. benthamiana* this was not possible due to virus-induced host mortality. These plants died after 6 weeks of infection, and therefore we were forced to collect tissue at this time point. As *D. stramonium* grows to similar heights as *N. tabacum* when infected with TEV, and *N. benthamiana* does not grow much after infection, we chose to maximize infection duration to make the results comparable to those obtained in *N. tabacum* [21]. We performed three 9-week passages in *D. stramonium* and – to keep the total evolutionary time comparable – five 6-week passages in *N. benthamiana*. In *D. stramonium* all ten lineages initiated were completed, whereas in *N. benthamiana* only 6/10 lineages were completed. The remaining four *N. benthamiana* lineages failed to cause infection in subsequent rounds of passaging, and were therefore halted. Initial symptomatology of TEV-eGFP in *N. benthamiana* was very mild, while this symptomatology was more severe in the second and subsequent passages, possibly indicating adaptation of the virus to this alternative host. In *D. stramonium* the symptomatology was constant along the evolution experiment.

Based only on previous results in *N. tabacum*, we expected that the exogenous *eGFP* gene sequence would be rapidly purged [21, 31, 32], and as such would serve as a first indicator of the occurrence of TEV adaptation. However, the usefulness of fluorescence for determining the integrity of the *eGFP* marker was limited in both hosts, by (*i*) the high levels of autofluorescence in the highly symptomatic *N. benthamiana* leaves, and (*ii*) the patchy fluorescence in the *D. stramonium* tissue. Therefore, unlike for TEV-eGFP in *N. tabacum*, the fluorescent marker was of limited use here. Nevertheless, all *N. benthamiana* lineages appeared to have some fluorescence until the end of the evolution experiment, and we observed a loss of fluorescence in only 1/10 *D. stramonium* lineages in the third 9-week passage.

**Fig. 2:**
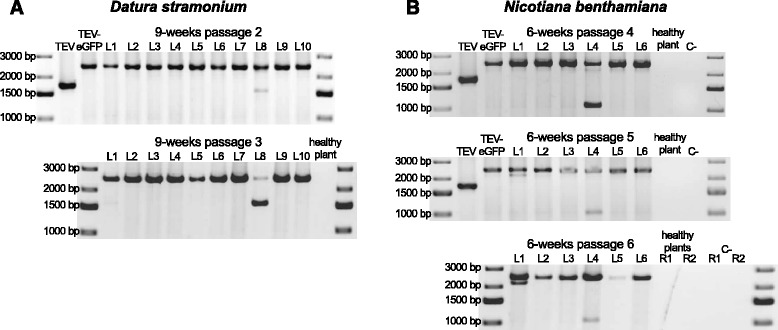
Deletion detection in the *eGFP* gene. Agarose gels with RT-PCR products of the region encompassing the *eGFP* gene. The TEV and TEV-eGFP are shown for comparative purposes. The negative controls are healthy plants and PCR controls (C-). **a** TEV-eGFP in *D. stramonium* has 10 independent lineages (L1-L10). A deletion encompassing the *eGFP* gene was detected in one lineage (L8) in the second 9-week passage. This deletion went to a high frequency in the subsequent passage. **b** TEV-eGFP in *N. benthamiana* has six independent lineages (L1-L6). A deletion bigger than the size of *eGFP* was detected in one lineage (L4) in the fourth 6-week passage. This deletion was not fixed in the two subsequent passages. A small deletion was detected in the fifth and sixth 6-week passage in L1

After each passage, RNA was extracted from the collected leaf tissue, and RT-PCR with primers flanking the *eGFP* insert was performed. This RT-PCR assay can therefore detect deletions in the *eGFP* gene, even when deletions extend well into the downstream *HC-Pro* gene [21]. In general, the RT-PCR results confirmed the fluorescence microscopy results: a large deletion was detected only in the one *D. stramonium* lineage with a loss of fluorescence (Fig. 2a; 9-weeks passage 2 L8). This deletion variant went to a high frequency in the subsequent passage (Fig. 2a; 9-weeks passage 3 L8). For *N. benthamiana* lineages, we did detect a low-frequency deletion in the *eGFP* gene in one lineage (Fig. 2b; 6-weeks passage 4 and 5 L4), but this deletion is so large that this variant is most likely no longer capable of autonomous replication. The deletion size is around 1500 nt, which means that besides deleting the entire *eGFP*, around 800 nt are deleted from HC-Pro, which has a size of 1377 nt in total. This deletion extends well into the central region of HC-Pro, beyond the well-conserved FRNK box, which is essential for virus movement and RNA-silencing suppressor activity [33, 34]. As complementation by full-length virus variants can slow down the rate at which deleterious variants are eliminated from the virus population [35], we performed an extra round of passaging with all *N. benthamiana* lineages to check whether this variant would remain at a low frequency, and found exactly this result (Fig. 2b; 6-week passage 6 L4). Furthermore, we detected a small deletion in one lineage (Fig. 2b; 6-week passage 5 and 6 L1) that was maintained at a low frequency in subsequent passages of the virus population.

### Whole-genome sequencing of the evolved lineages

All evolved and the ancestral TEV-eGFP lineages were fully sequenced by Illumina technology (SRA accession: SRP075180). The consensus sequence of the ancestral TEV-eGFP population was used as a reference for mapping the evolved lineages. The deletion observed by RT-PCR (Fig. 2a) in one of the *D. stramonium* lineages was confirmed by a low number of reads mapping inside the *eGFP* gene (median coverage: 111.5), compared to a higher average coverage outside this region (median coverage *P1* gene: 19190, median overall genome coverage: 18460). The large deletion included the N-terminal region of *HC-Pro*, as observed for other deletions that occur after gene insertions before this gene [21, 36]. For all other lineages in *D. stramonium* and *N. benthamiana*, coverage over the genome was largely uniform and similar to the ancestral virus population, indicating that there were indeed no genomic deletions present at appreciable frequencies.

**Fig. 3:**
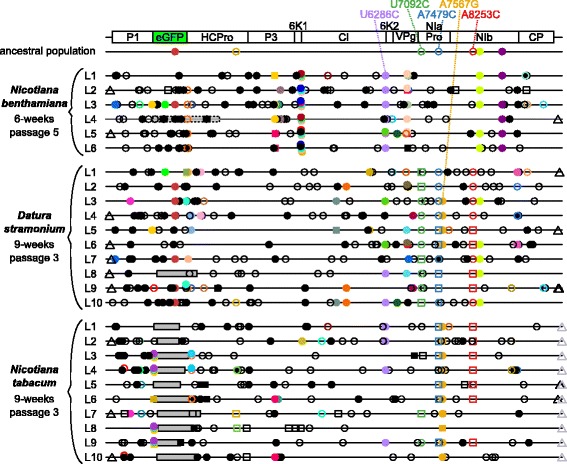
Genomes of the TEV-eGFP lineages evolved the three different hosts as compared to the ancestral lineage. Mutations were detected using NGS data of the evolved lineages (L1-L10), as compared to their ancestral population. The square symbols represent mutations that are fixed (>50%) and the circle symbols represent mutations that are not fixed (<50%). Filled symbols represent nonsynonymous substitutions and open symbols represent synonymous substitutions. The triangle symbols represent mutations that are present in either the 3’UTR or 5’UTR. *Black* substitutions occur only in one lineage, whereas color-coded substitutions are repeated in two or more lineages. Note that the mutations are present at different frequencies as reported by VarScan 2. *Grey* boxes with continuous *black* lines indicate genomic deletions in the majority variant of the virus population. The grey transparent box with dotted *black* lines in L4 of *N. benthamiana* indicates a genomic deletion in a minority variant. The latter box was drawn to indicate the size of the deletion, assuming that the deletion starts at the first position of *eGFP*. The mutations discussed in the manuscript are highlighted on the top. For more information on the frequency of the mutations please see Additional file 2: Tables S1-S3

Single nucleotide mutations were detected from a frequency as low as 1%, comparing the evolved TEV-eGFP lineages in *N. benthamiana* and *D. stramonium* to the ancestral population (Fig. 3). This detection was also performed for evolved TEV-eGFP lineages in *N. tabacum*, that were sequenced in a previous study [21] (SRA accession: SRP075228). In the evolved *N. benthamiana* lineages 165 unique mutations were found, with a median of 34.5 (range: 27–47) mutations per lineage. In the evolved *D. stramonium* lineages 239 unique mutations were found, with a median of 31.5 (range: 16–35) mutations per lineage. In the evolved *N. tabacum* lineages, 183 unique mutations were found, with a median of 21.5 (range: 17–36) mutations per lineage. Note that fixed single nucleotide mutations (frequency > 50%) are also detected in the evolved lineages (squared symbols in Fig. 3), as the detection was done over the ancestral population. Hence, it allows us to compare the mutations that arose by evolving TEV-eGFP in the different hosts.

We detected only one mutation (U6286C; CI/Y2096H) that is shared between all three hosts. However, this mutation was present at a low frequency and not detected in all *D. stramonium* and *N. tabacum* lineages (Fig. 3 and Table 1). The *N. benthamiana* and *D. stramonium* lineages share more mutations (15) than either *N. benthamiana* or *D. stramonium* share with *N. tabacum* (4 and 9, respectively). However, most of these mutations are present in only a few lineages and at low frequency (Fig. 3 and Table 1).

**Table 1:** TEV-eGFP mutations shared in the different hosts

			*N. benthamiana*		*D. stramonium*		*N. tabacum*	
Nucleotide change	Amino acid change	Gene	Number of lineages	Frequency range	Number of lineages	Frequency range	Number of lineages	Frequency range
U6286C	Y2096H	*CI*	6/6	0.013 – 0.131	2/10	0.012 – 0.031	4/10	0.019 – 0.140
A208G	M70V	*P1*	1/6	0.012	1/10	0.010	-	-
C1039U	H347Y	*P1*	1/6	0.013	1/10	0.035	-	-
G1332A	M444I	*eGFP*	1/6	0.176	1/10	0.139	-	-
U1556G^a^	V519G	*eGFP*	1/6	0.011	6/10	0.010 – 0.016	-	-
U1836G	synonymous	*HC-Pro*	5/6	0.093 – 0.108	1/10	0.089	-	-
A1917G	synonymous	*HC-Pro*	1/6	0.015	1/10	0.017	-	-
A6278G	E2093G	*CI*	1/6	0.012	2/10	0.014 – 0.031	-	-
C6547U	H2183Y	*VPg*	1/6	0.013	1/10	0.014	-	-
U6747C	synonymous	*VPg*	1/6	0.012	1/10	0.110	-	-
A6776G	D2259G	*VPg*	2/6	0.533 – 0.746	1/10	0.023	-	-
G6803A	S2268N	*VPg*	1/6	0.014	1/10	0.024	-	-
A6438G	synonymous	*6 K2*	1/6	0.012	1/10	0.024	-	-
C8405G^a^	T2802R	*NIb*	5/6	0.010 – 0.018	5/10	0.010 – 0.016	-	-
U9474C	synonymous	*CP*	1/6	0.013	1/10	0.061	-	-
C9837U	synonymous	*CP*	1/6	0.070	1/10	0.010	-	-
U3803C	I1268T	*P3*	2/6	0.656 – 0.881	-	-	2/10	0.010 – 0.019
U3872C	V2191A	*P3*	1/6	0.064	-	-	1/10	0.016
G4411A	V1471I	*CI*	1/6	0.030	-	-	1/10	0.016
C4989U	synonymous	*CI*	1/6	0.018	-	-	1/10	0.066
C548U	T183I	*P1*	-	-	1/10	0.011	1/10	0.024
G2928A^a^	synonymous	*HC-Pro*	-	-	1/10	0.017	1/10	0.999
U7092C^a^	synonymous	*NIa-Pro*	-	-	10/10	0.091 – 0.755	2/10	0.176 – 0.999
A7479C^a^	synonymous	*NIa-Pro*	-	-	10/10	0.132 – 0.790	7/10	0.960 – 0.999
A7567G	K2523E	*NIa-Pro*	-	-	4/10	0.012 – 0.053	10/10	0.015 – 0.871
G7710A	synonymous	*NIa-Pro*	-	-	1/10	0.014	1/10	0.024
A8253C^a^	synonymous	*NIb*	-	-	10/10	0.136 – 0.801	7/10	0.805 – 0.998
G9117A	synonymous	*NIb*	-	-	1/10	0.321	2/10	0.022 – 0.025
U9249C	synonymous	*NIb*	-	-	2/10	0.011 – 0.231	1/10	0.040

^a^Also detected in the ancestral population

The synonymous mutations U7092C, A7479C and A8253C, that are shared between *D. stramonium* and *N. tabacum*, are present in the highest number of lineages and reach higher frequencies among all shared mutations detected in the three hosts. These mutations were already present in the ancestral population, nevertheless, the frequencies at which these mutations are present display interesting patterns. In both *D. stramonium* and *N. tabacum* the mutations A7479C and A8253C are always detected at the same frequency within each lineage, suggesting a strong linkage between them (Additional file 1: Fig. S1). Furthermore, the U7092C mutation never appears together with the former two mutations (Additional file 1: Fig. S1), suggesting that this mutation occurs in another haplotype. Interestingly, the ancestral U7092C, A7479C and A8253C mutations were not detected in the *N. benthamiana* lineages, demonstrating the differences in host-pathogen interactions. Moreover, mutation A7567G, which was not present in the ancestral population, also appears only in the *D. stramonium* and *N. tabacum* lineages (Additional file 1: Fig. S1).

Host-specific mutations were mostly found in the evolved TEV-eGFP lineages of *N. benthamiana* (Fig. 3 and Table 2). In this host, a total number of 7 specific mutations were detected, all of them being nonsynonymous. In *D. stramonium* no host-specific mutations were detected. In *N. tabacum* only one host-specific mutation was detected in the 3’UTR (Table 2). Note that host specific mutations were defined as mutations detected in at least half of the evolved lineages. For more information on the mutations found in the three hosts please see Additional file 2: Tables S1-S3.

**Table 2:** Host specific mutations in the evolved TEV-eGFP lineages

	Nucleotide change	Amino acid change	Gene	Number of lineages	Frequency range
*N. benthamiana*	G3797A	G1266E	*P3*	3/6	0.291 – 0.664
	G4380U	E1460D	*6K1*	3/6	0.012 – 0.093
	U4387C	Y1463H	*6K1*	4/6	0.011 – 0.016
	C4391U	T1464M	*6K1*	6/6	0.041 – 0.138
	G4397A	S1466N	*CI*	6/6	0.012 – 0.019
	A6771U	L2257F	*VPg*	4/6	0.027 – 0.201
	G8909U^a^	W2970L	*NIb*	5/6	0.026 – 0.042
*D. stramonium*	-	-	-	-	-
*N. tabacum*	G10253A		3’UTR	10/10	0.025 – 0.040

^a^Also detected in the ancestral population

### Viral accumulation and within-host competitive fitness

**Fig. 4:**
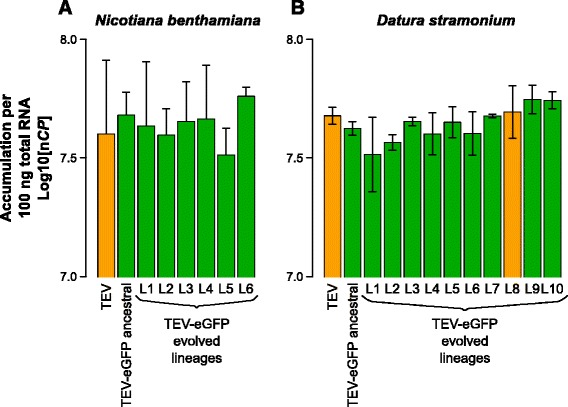
Virus accumulation of the evolved and ancestral lineages. Virus accumulation, as determined by accumulation experiments and RT-qPCR at 10 dpi, of TEV, the ancestral TEV-eGFP, and the evolved TEV-eGFP lineages in *N. benthamiana* (**a**) and *D. stramonium* (**b**). TEV and the evolved lineage with a deletion in the *eGFP* gene (B; L8) are indicated with the *orange* bars. The ancestral TEV-eGFP and the evolved lineages with an intact *eGFP* gene are indicated with the *green* bars. No significant differences between TEV and TEV-eGFP ancestral were found, nor between these and the evolved lineages (*t*-test with Holm-Bonferroni correction for multiple tests). Error bars represent SD of the plant replicates

We measured virus accumulation 10 dpi, by RT-qPCR for a region within the coat protein gene (*CP*). In both host species, we found no statistically significant differences (*t*-test with Holm-Bonferroni correction) between TEV, TEV-eGFP and the lineages of TEV-eGFP evolved in that host (Fig. 4).

**Fig. 5:**
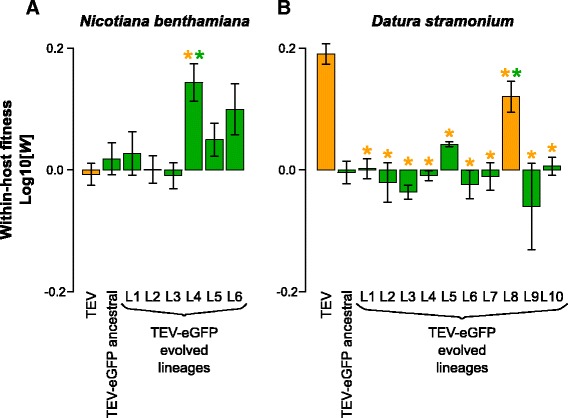
Within-host competitive fitness of the evolved and ancestral lineages. Fitness (*W*) determined by competition experiments and RT-qPCR of the different viral genotypes with respect to a common competitor; TEV-mCherry. *W* was determined at 10 dpi, of TEV, the ancestral TEV-eGFP, and the evolved TEV-eGFP lineages in *N. benthamiana* (**a**) and *D. stramonium* (**b**). TEV and the evolved lineage with a deletion in the *eGFP* gene (B; L8) are indicated with the *orange* bars. The ancestral TEV-eGFP and the evolved lineages with an intact *eGFP* gene are indicated with the *green* bars. The *orange* asterisks indicate statistical significant differences of the evolved lineages as compared to TEV (*t*-test with Holm-Bonferroni correction for multiple tests). The *green* asterisks indicate statistical significant differences of the evolved lineages as compared to the ancestral TEV-eGFP (*t*-test with Holm-Bonferroni correction for multiple tests). Not indicated in the figure is that TEV and TEV-eGFP ancestral tested significantly different in *D. stramonium* (B; *t*-test: *t*_4_ = 13.438, *P* <0.001) while not in *N. benthamiana* (A; *t*-test: *t*_4_ = −1.389, *P* = 0.237). Error bars represent SD of the plant replicates

We then measured within-host competitive fitness by means of head-to-head competition experiments with TEV-mCherry, a virus with a different marker but similar fitness to TEV-eGFP [23]. When serially passaging a virus in a single host plant, the outcome of this assay probably better reflects viral fitness than accumulation, because the relative and not absolute numbers of virus variants present will determine which variants dominate [21]. Here we observed interesting differences between TEV and TEV-eGFP in the two different hosts. Whereas the TEV-eGFP had lower fitness than the wild-type virus in *D. stramonium* (Fig. 5b, compare TEV and ancestral TEV-eGFP; *t*-test: *t*_4_ = 13.438, *P* <0.001), there was no difference in *N. benthamiana* (Fig. 5b, compare TEV and ancestral TEV-eGFP; *t*-test: *t*_4_ = −1.389, *P* = 0.237). Our results therefore suggest that although there is a fitness cost associated with the *eGFP* gene in *N. tabacum* [21] and *D. stramonium,* there is none in *N. benthamiana*. Interestingly, *N. tabacum* and *N. benthamiana* are more closely related to each other than either species is to *D. stramonium*, and yet the host species has a strong effect on the costs of a heterologous gene. This observation clearly clashes with our expectation that *eGFP* would have a fitness cost.

For only 1/10 of the evolved lineages in *D. stramonium*, we observed a significant increase in competitive fitness compared to the ancestral TEV-eGFP (Fig. 5b, L8; *t*-test with Holm-Bonferroni correction: *t*_4_ = −6.890, *P* = 0.002). This lineage is the only one to have a deletion in the *eGFP* insert. In *N. benthamiana*, 1/6 lineages had a significant increase in within-host fitness (Fig. 5a, L4; *t*-test with Holm-Bonferroni correction: *t*_4_ = −5.349, *P* = 0.006). However, this increase in fitness probably is not associated with the large genomic deletion for three reasons: (*i*) the wild-type TEV without the *eGFP* gene has a similar fitness compared to the ancestral TEV-eGFP, suggesting deletions in *eGFP* would not be beneficial, (*ii*) the RT-PCR results show that this variant occurs at a low frequency in the population, and therefore is unlikely to affect strongly the results of the competition assay, and (*iii*) this deletion variant remains at low frequency during the next round of passaging (Fig. 2b), suggesting that while frequency-dependent selection might occur, its fitness is not higher than the coevolving full-length TEV-eGFP. Moreover, another lineage of *N. benthamiana* where we did not detect any deletions, also appeared to have increased in fitness (Fig. 5a, L6; *t*-test: *t*_4_ = −4.0792, *P* = 0.015), however, after the Holm-Bonferroni correction not significantly. Interestingly, the lineage that did increase its fitness significantly (L4) is the only lineage that contains mutations in the 6 K2 protein in this host (Additional file 2: Table S1). Therefore, we speculate that single-nucleotide variation is one of the main driving forces for an increase in TEV-eGFP fitness in *N. benthamiana*.

These fitness measurements show that most lineages failed to adapt to the new host species. However, in the two cases that there were significant fitness increases, the underlying genetic changes were consistent with the expected route of adaptation, when our expectations were modified based on the unexpected results for the competitive fitness of the ancestral TEV-eGFP virus in the two alternative host species. In *D. stramonium*, where carrying *eGFP* imposes a high fitness cost, this sequence was deleted. In *N. benthamiana*, where carrying *eGFP* apparently has not fitness cost, host-specific single-nucleotide variation was observed.

## Discussion

We set out to explore the hypothesis that differences in virulence for different hosts could have an effect on the rate of virus adaptation in each host [13]. Although we find this hypothesis simple and provocative, the observed patterns in our experiments suggest that even in a controlled laboratory environment, the biological reality will often be complex and hard to predict. We used a virus expressing an eGFP fluorescent marker in the hope that the loss of this marker could serve as a real-time indicator of adaptation. Even though this method served us in the past in the natural host *N. tabacum* [21], there were complications with this method in the two alternative hosts used in this study, and a loss of fluorescence was only observed in a single *D. stramonium* lineage. RT-PCR and Illumina sequencing confirmed the loss of the eGFP marker in this case, and its integrity in all other lineages. The data of our competitive fitness assay demonstrate why the marker sequence was probably rather stable in *N. benthamiana*; *eGFP* does not appear to have a cost in this host species, in sharp contrast to the strong fitness cost observed in *D. stramonium* as well as previously observed in the more closely related host *N. tabacum* [21]. We expect that the marker will eventually be lost, but only due to genetic drift and therefore at a slow rate. The observed lack of fitness cost for *eGFP* in *N. benthamiana* was an unexpected result, and further undermined our initial idea of using the loss of this gene as a real-time indicator of virus adaptation.

What mechanisms might underlie the difference in the fitness costs of *eGFP* marker in these two host plants? Does the difference in passage length used influences the rate of marker loss in *N. benthamiana* and *D. stramonium*? In a previous study, we showed that the loss of the *eGFP* marker occurred more rapidly as the duration of each passage was increased [21]. During long passages transmission bottlenecks are more spaced in time, and much larger census population sizes are reached. Moreover, longer passages also give a much greater opportunity for virus movement into the newly developing host tissues. This does not apply to *N. benthamiana*; due to the high virulence of TEV, these plants did not grow much and eventually died. During passaging of TEV-eGFP, leaves were therefore harvested after 6 weeks, just before plants were completely dead. We think that the marked differences in virus-host interactions will be the main determinants of virus evolution and marker stability, although we cannot rule out categorically that the differences in passage length also might have played a role. As for *N. tabacum* [21], here we again observed that the eGFP marker does not affect virus accumulation, whereas it does lower competitive fitness in *D. stramonium*. These observations suggest that the effects of the eGFP marker on virus movement are the main reason for selection against the marker, as for TEV cellular co-infections are rare and therefore fast within-host spread is crucial to invade more space and hereby having a higher competitive fitness [37]. However, marker loss in *D. stramonium* appears to occur more slowly compared to *N. tabacum* [21], indicating poor virus adaptation to this alternative host. Given the high virulence of TEV for *N. benthamiana*, including strong stunting, there will be limited virus movement during infection and thus significantly lower population census sizes. Hence, we speculate that in *N. benthamiana* the limited scope for virus movement and accumulation – due to the virus’ virulence itself – might mitigate the cost of the eGFP marker. Alternatively, cell-to-cell and systemic virus movement in *N. benthamiana* might be so slow that the addition of the eGFP marker matters little. A slow systemic virus movement may also explain why in the second round of infection four lineages failed to re-infect *N. benthamiana*, as initial virus accumulation appeared to be very low until possible virus adaptation by means of point mutations occurred.

These results are at odds with our expectations, but they nevertheless have some interesting implications. First, host species changes can apparently ameliorate the costs of exogenous genes. Although strong virus genotype-by-host species interactions have been previously shown for TEV [17], we did not anticipate that a such a simple difference (the presence of *eGFP*) could also be subjected to such an interaction. These results suggest that when considering the evolution of genome architecture, host species might play a very important role, by allowing evolutionary intermediates to be competitive. For example, for TEV we have shown that the evolution of an alternative gene order through duplication of the *NIb* replicase gene is highly unlikely, as this intermediate step leads to significant decreases in fitness, making the trajectory to alternative gene orders inaccessible [36]. We have also shown that potentially beneficial gene duplications in the TEV genome are selected against, as these also lead to a significant reduction in viral fitness [38]. If gene duplication has a similar interaction with host species as the *eGFP* insert has, then an alternative host species could act as a stepping-stone and hereby increase the accessibility of the evolutionary trajectories to alternative genome architectures. Similar effects of environmental change have been noted in other studies [39]. The generality of these results has not been addressed yet using other viruses with altered genome architecture, but the possibilities are tantalizing. Second, our results could also have implications for assessing the biosafety risks of the genetically modified organisms. Our results suggest that extrapolating fitness results from a permissive host to alternative hosts can be problematic, even when the scope for unexpected interactions appears to be limited, as would be the case for the addition of eGFP expression. In other model systems, unexpected interactions between heterologous genes and host species have also been reported [40].

Upon passaging in each alternative host species, only a single lineage evolved higher fitness. The low rate of adaptation observed was consistent with a previous report [18], although we used passages of a longer duration here and had therefore expected more rapid adaptation [21]. Given the low rate at which lineages adapted in this experiment, however, although the rate of adaptation appears to be similar in both hosts, we do not consider that our results allow for a good test of our hypothesis. Nevertheless, our results do stress that differences in host biology can have a much stronger effect on evolutionary dynamics than differences in virus-induced virulence between host species. An alternative way to tackling the question of the effects of virulence on adaptation might be to use a biotechnological approach; hosts which have different levels of virulence can be engineered, to ensure the main difference between host treatment is microparasite-induced virulence. For example, plant hosts could be engineered to express antiviral siRNAs at low levels. Such an approach would allow for a more controlled test of the hypothesis suggested here, whilst probably not being representative for natural host populations. On the other hand, such experiments could perhaps help shed light on the effects of virulence on adaptation in agroecosystems or vaccinated populations.

## Conclusions

A host species jump can be a game changer for evolutionary dynamics. An exogenous sequence – *eGFP* – which is unstable in its typical host, has shown to be more stable in two alternative host species for which TEV has both lower and higher virulence than in the typical host. In addition, *eGFP* does not appear to have any fitness effects in the host for which TEV has high virulence. These observations clashed with the hypothesis that high virulence slows down the rate of adaptation. Moreover, when considering the evolution of genome architecture, host species jumps might play a very important role, by allowing evolutionary intermediates to be competitive.

## Additional files

Additional file 1: Figure S1. Frequency of mutations found in both *D. stramonium* and *N. tabacum*. Mutations detected in both *D. stramonium* and *N. tabacum* that were present in all the lineages of either one of these hosts. The frequency of these mutations in either the ancestral population (anc) or the different lineages (L1-L10) is given by the color-coded points. (PDF 44 kb)
Additional file 2: Table S1. Mutations detected in the *N. bentiamiana* lineages as compared to the ancestral lineage. **Table S2.** Mutations detected in the *D. stramonium* lineages as compared to the ancestral lineage. **Table S3.** Mutations detected in the *N. tabacum* lineages as compared to the ancestral lineage. (XLS 149 kb)
