# Supplementary figures and images for: High virulence does not necessarily impede viral adaptation to a new host: a case study using a plant RNA virus
Source: BMC Evol Biol. 2017 Jan 19;17:25. doi: 10.1186/s12862-017-0881-7 (PMC5248479; doi:10.1186/s12862-017-0881-7)

**A***Datura stramonium*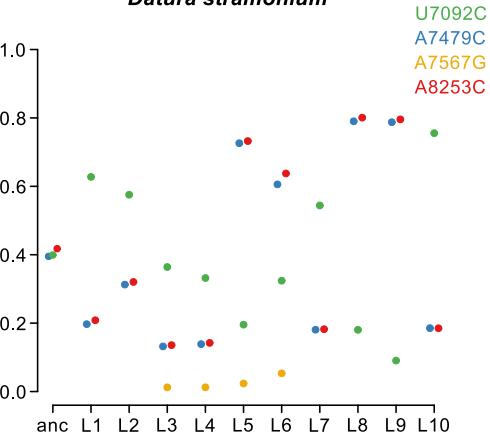**B***Nicotiana tabacum*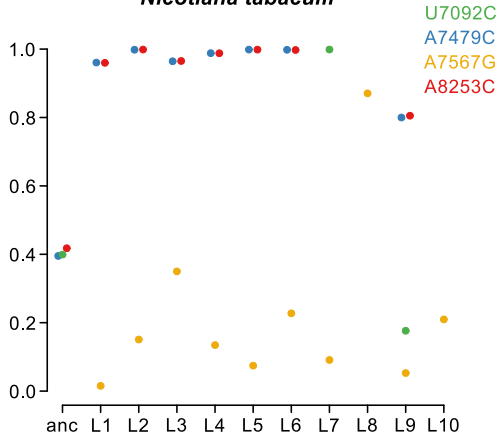

Supplement: Additional file 1: Figure S1. — Frequency of mutations found in both D. stramonium and N. tabacum. Mutations detected in both D. stramonium and N. tabacum that were present in all the lineages of either one of these hosts. The frequency of these mutations in either the ancestral population (anc) or the different lineages (L1-L10) is given by the color-coded points. (PDF 44 kb) [file 12862_2017_881_MOESM1_ESM.pdf]
